# Determinants of the acceptance and adoption of a digital contact tracing tool during the COVID-19 pandemic in Singapore

**DOI:** 10.1017/S0950268822000401

**Published:** 2022-03-02

**Authors:** Zhilian Huang, Huiling Guo, Hannah Yee-Fen Lim, Angela Chow

**Affiliations:** 1 Department of Clinical Epidemiology, Office of Clinical Epidemiology, Analytics, and Knowledge (OCEAN), Tan Tock Seng Hospital, Singapore, Singapore; 2 Nanyang Business School, Nanyang Technological University, Singapore, Singapore; 3 Lee Kong Chian School of Medicine, Nanyang Technological University, Singapore, Singapore

**Keywords:** Digital contact tracing tool, contact tracing, COVID-19 pandemic, technology acceptance and adoption, smartphone apps

## Abstract

The motivations that govern the adoption of digital contact tracing (DCT) tools are complex and not well understood. Hence, we assessed the factors influencing the acceptance and adoption of Singapore's national DCT tool – TraceTogether – during the COVID-19 pandemic. We surveyed 3943 visitors of Tan Tock Seng Hospital from July 2020 to February 2021 and stratified the analyses into three cohorts. Each cohort was stratified based on the time when significant policy interventions were introduced to increase the adoption of TraceTogether. Binary logistic regression was preceded by principal components analysis to reduce the Likert items. Respondents who ‘perceived TraceTogether as useful and necessary’ had higher likelihood of accepting it but those with ‘Concerns about personal data collected by TraceTogether’ had lower likelihood of accepting and adopting the tool. The injunctive and descriptive social norms were also positively associated with both the acceptance and adoption of the tool. Liberal individualism was mixed in the population and negatively associated with the acceptance and adoption of TraceTogether. Policy measures to increase the uptake of a national DCT bridged the digital divide and accelerated its adoption. However, good public communications are crucial to address the barriers of acceptance to improve voluntary uptake widespread adoption.

## Introduction

Contact tracing is an essential strategy for mitigating the transmission of SARS-CoV-2 and reducing the morbidity and mortality associated with COVID-19 [1]. Effective contact tracing is dependent on the speed of identifying and isolating persons who are susceptible to contracting an infectious disease from exposure to an infectious agent. However, the scale of the COVID-19 pandemic limited the capacity of the labour-intensive contact tracing process. Many countries had to implement lockdowns to halt the transmission of SARS-CoV-2 to prevent overburdening the health system [2].

Digital contact tracing (DCT) tools, available as a smartphone app or wearable device, can complement contact tracing activities during a pandemic when implemented at scale [3]. For example, the time taken for contact tracing can be 2.5 times shorter when a sizeable proportion of the population (i.e. minimally 60%) adopts a DCT tool when engaging in social activities [4–6]. DCT tools can also reduce recall biases and identify physical close-proximity contacts outside the infected person's social circles [7]. Despite the potential of DCT tools in enhancing contact tracing capabilities, its implementation is fraught with challenges associated with and beyond technology adoption [8].

One challenge associated with technology adoption is the accessibility of DCT tools for the masses. A high smartphone penetration rate is a prerequisite for successfully implementing mobile phone applications (app) based DCT tools in the population [3]. Singapore distributed Bluetooth-enabled wearable tokens to its entire population despite a high smartphone penetration rate of 88% to ensure complete accessibility of its national DCT tool – TraceTogether – for all residents [9, 10]. However, removing barriers to the accessibility of a DCT tool does not address the many challenges of technology adoption. As illustrated by the diffusion of innovation theory, there is a lag time from implementation to innovation adoption due to the varying pace of technology acceptance and adoption within a population [11]. Yet, the need to rapidly scale up the adoption of DCT tools is vital in a pandemic response and policy interventions are required to rapidly increase adoption rates [12].

Challenges beyond technology adoption include knowledge deficits pertaining to the DCT tool's operational system and misunderstanding of the laws that govern the data privacy and protection of personal data [13–15]. Fears of data mishandling, concerns on personal data breach, and mistrust in the government have led to hesitancy in the adoption of DCT tools [14, 16, 17]. Widespread social media use has also enabled the mass dissemination of pessimistic or misleading information on DCT tools that could intensify feelings of hesitancy towards its adoption [16, 18]. Despite the hesitancy in DCT tool adoption, the notions of the common good may have driven the individual to adopt practices that are beneficial to society during a pandemic [12, 19].

The adoption rates of Singapore's national DCT tool – TraceTogether – increased from 20% in May 2020 to more than 70% by December 2020 after a slew of policy and public education measures (i.e. distribution of wearable tokens, mandatory check-ins at public venues, mass media communication) to increase adoption rates [9]. As the concept of DCT tools is unprecedented before the COVID-19 pandemic, the complex motivations that govern the adoption of such tools have not been previously studied and are not well understood. Hence, we aimed to assess the factors influencing the acceptance and adoption of TraceTogether during the COVID-19 pandemic, anchoring on established behavioural models.

## Methods

### Study design

We conducted a serial cross-sectional study to assess the awareness, acceptance and perceptions on the use of TraceTogether from 6 July 2020, through 26 February 2021. The study period stretched across the various phases of the COVID-19 pandemic during which multiple national policy measures were implemented to increase the adoption of TraceTogether. This enabled us to assess changes in the attitudes and perceptions of TraceTogether at different phases of the pandemic. Respondents were stratified by four age categories and gender to ensure a proportionate and diverse respondent coverage.

### Study setting

We recruited patients and visitors of Tan Tock Seng Hospital's two busiest outpatient clinics. Each clinic sees (up to 400) patients per working day on average. Tan Tock Seng Hospital is the second largest public hospital in Singapore and services a resident population of 1.4 million.

### Questionnaire design

The survey instrument was developed based on the constructs of various extended Technology Acceptance models corresponding to health technology acceptance. Davis's Technology Acceptance Model (TAM) posits that the perceived usefulness and ease of use of technology influences the acceptance and eventual adoption of a new technology. Several scholarly works have extended the TAM by integrating health behavioural models or constructs related to consumers' health-seeking behaviour to explain the process of health technology adoption. For example, Beldad *et al*. extended the TAM with the inclusion of trust in the app developer, the injunctive and descriptive social norms, and health valuation to determine the factors influencing German users' willingness to continue using a fitness app [20]. The Health Information Technology Acceptance Model (HITAM) considers constructs from the Health Belief Model and Theory of Planned Behaviour [21]. Other studies also considered the perceived security of a wearable device [22] and included demographic variables in predicting health technology adoption. In addition, the notions of the common good may override liberal individualism in promoting the uptake of beneficial health behaviour during the protracted COVID-19 pandemic [19, 23].

The final questionnaire included 23 five-point Likert scale questions based on the constructs of TAM, injunctive social norms, descriptive social norm, trust in DCT tool developer, health valuation, the perceived security of the DCT tool and liberal individualism to assess the attitudes and perceptions of respondents on the adoption of TraceTogether (Supplementary Table S1). The questionnaire was translated to Mandarin and the Malay language and piloted with 154 individuals across gender, age and ethnic groups, and educational levels. The wording of some questions were revised for better clarity and understanding, based on respondents' feedback.

### Data collection

The survey was interviewer-administered to minimise misinterpretations and to overcome language barriers among the elderly population. The interviewers were trained to administer the questions in a standardised manner. Respondents were asked if they were using the ‘TraceTogether’ app or token, their willingness to use the ‘TraceTogether’ tool, followed by the Likert scale questions on their attitudes and perceptions on the use of TraceTogether during the COVID-19 pandemic. Demographic information was collected to adjust for potential confounding due to demographic factors in the model. We estimated that a minimum sample size of 816 per cohort was required to detect all differences with effect sizes of ≥1.3 in the attitudes and perceptions between the adopters and non-adopters of a DCT tool, with ≥50% prevalence of the attitude/perception among adopters, at 80% power and 5% significance level.

### Outcome

The outcomes of interest are the acceptance of TraceTogether (whether the respondent is willing to use TraceTogether) and adoption of TraceTogether (whether the respondent was using TraceTogether at the point of the survey).

### Data stratification

We stratified the analyses into three cohorts based on the time period when significant policy interventions were introduced to increase the adoption of TraceTogether. The first cohort was stratified by responses collected from July 2020 to October 2020. This period corresponds to the announcement and national distribution of TraceTogether tokens to increase its accessibility and encourage its adoption. The second cohort, stratified by responses collected from November 2020 to December 2020, corresponds to announcements on mandatory TraceTogether check-ins to public venues and the easing of social restrictions when TraceTogether attains a minimum adoption rate of 70% nationwide. Although the announcement of mandatory TraceTogether check-ins was made on 20 October 2020, we expected that an amount of time would be required for the public to adjust to the new measures. The third cohort was stratified by responses collected from January 2021 to February 2021. TraceTogether uptake had exceeded 70% by December 2020, but news that the data collected by TraceTogether would be used for criminal investigations of serious crimes sparked a public outcry and evoked negative sentiments towards the use of TraceTogether.

### Statistical analysis

#### Factor analysis

We conducted Principal Component Analysis (PCA) to reduce the Likert scale items. An eigenvalue of >1 was used to determine the optimal number of factors for dimension reduction. Variables were removed in a stepwise manner until no variable (1) with a factor loading higher than 0.5 loads on more than one factor and (2) had the highest factor loading of less than 0.5. The variable removal at each step was checked to ensure that there was no reduction in the variance explained. Internal consistencies were assessed by computing the Cronbach's *α* for each factor. A Cronbach's *α* score of >0.7 is generally considered good.

#### Logistic regression

Finally, we used multivariable logistic regression models to explore the independent factors associated with the acceptance and adoption of TraceTogether for each cohort. The model with the lowest Akaike's information criterion value was preferred, but the inclusion of statistically significant variables of interest was prioritised in the final models (Supplementary Table S2). We entered variables into the model in a stepwise manner. First, we included all the factors derived from PCA in the model, followed by the variables removed from the PCA and demographic variables such as age group, education level, gender and employment status of the respondent. We also checked the variance inflation factor to ensure that all the regression models were not affected by multicollinearity and ensured that all statistical assumptions were valid. IBM SPSS Statistics for Windows, version 26 (IBM Corp., Armonk, NY, USA) [24] was used for all statistical analyses.

## Results

### Characteristics of respondents

The age and gender of 3943 respondents were evenly distributed from the purposive sampling. One-third (31.3%) of respondents were tertiary educated and two-thirds (66.4%) were employed with a full-time or part-time job. The proportions of tertiary educated and employed respondents remained the same over the cohorts, signifying an even cohort stratification (Table 1).

**Table 1.** Baseline characteristics of respondents

	Total	Cohort 1 (Jul–Oct 2020)	Cohort 2 (Nov–Dec 2020)	Cohort 3 (Jan–Feb 2021)
	(*N* = 3943) *n* (%)	(*N* = 1812) *n* (%)	(*N* = 1274) *n* (%)	(*N* = 857) *n* (%)
Sex				
Male	1970 (50.0%)	891 (49.2%)	653 (51.3%)	426 (49.7%)
Age (21–80 years old)				
Mean (s.d.)	50.2 (16.8)	49.5 (16.6)	50.9 (17.0)	50.5 (17.0)
Education level				
Tertiary	1235 (31.3%)	586 (32.3%)	383 (30.1%)	266 (31.0%)
Employment status^a^				
Employed	2617 (66.4%)	1231 (67.9%)	836 (65.6%)	550 (64.2%)
Acceptance of TraceTogether				
Willing to use TraceTogether	3037 (77.0%)	1236 (68.2%)	1061 (83.3%)	740 (86.3%)
Adoption of TraceTogether				
Using TraceTogether	2238 (56.8%)	695 (38.4%)	814 (63.9%)	729 (85.1%)

a The not employed status includes respondents who are unemployed, retired, under military service and full-time students.

The proportion of respondents who were willing to use TraceTogether increased from an average of 68.2% in cohort 1 (July 2020 to October 2020), to 83.3% in cohort 2 (November 2020 to December 2020) and 86.3% in cohort 3 (January 2021 to February 2021). Likewise, the proportion of respondents who were using TraceTogether increased rapidly from 38.4% in cohort 1, to 63.9% in cohort 2 and 85.1% in cohort 3 (Table 1).

**Fig. 1.**
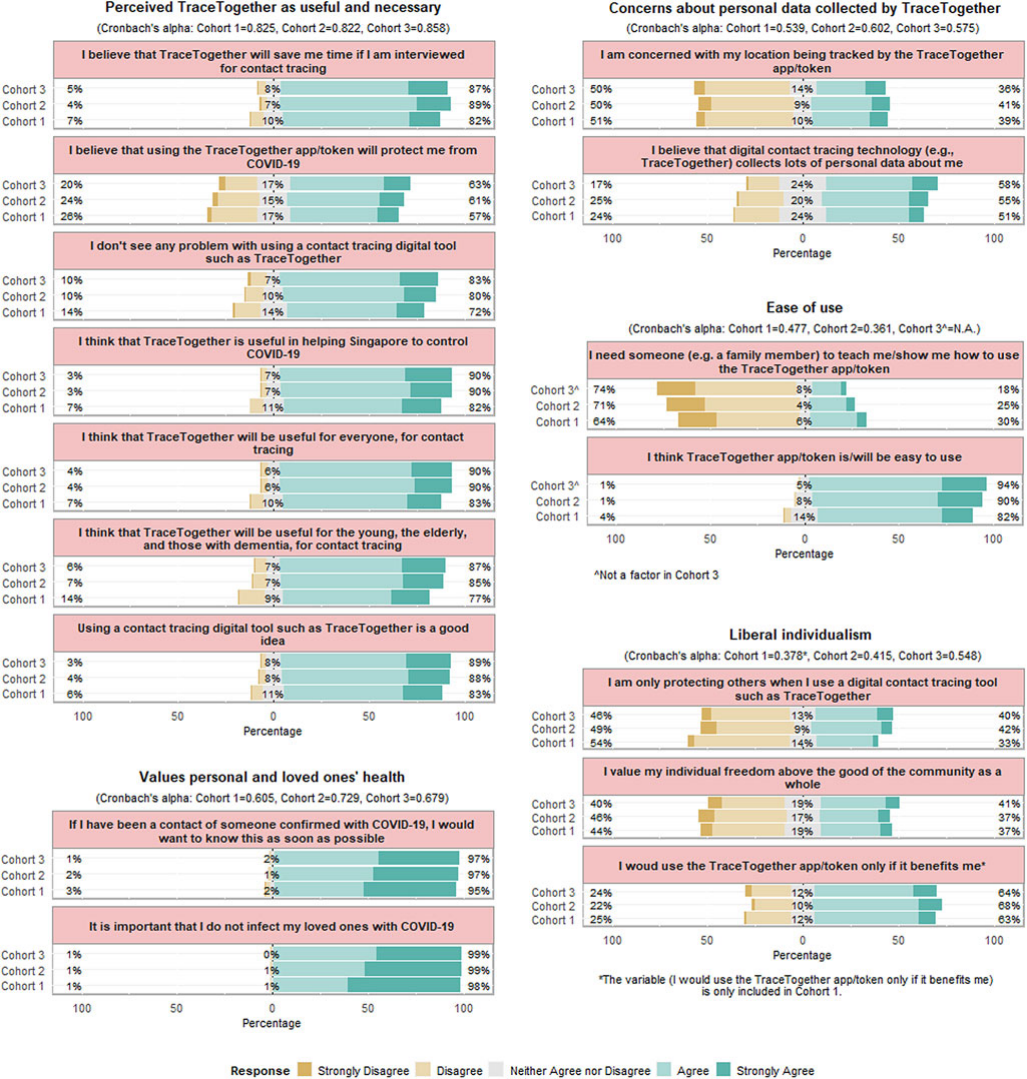
Anchored divergent plot of factors influencing the acceptance and adoption of TraceTogether during the COVID-19 pandemic. The dataset was stratified into three cohorts based on the time when significant policy interventions were introduced to increase the adoption of TraceTogether. Cohort 1 corresponds to responses collected from July 2020 to October 2020, cohort 2 from November 2020 to December 2020 and cohort 3 from January 2021 to December 2021.

PCA reduced 60–70% of the Likert scale statements into five factors (Fig. 1). Three factors, ‘Perceived TraceTogether as useful and necessary’, ‘Values personal and loved ones’ health’ and ‘Concerns about personal data collected by TraceTogether’, emerged in all three cohorts. Most respondents (>80%) agreed that TraceTogether was useful and necessary, and they valued their and their loved ones' health in the pandemic.

Approximately half of the respondents had reservations about the type of data collected by TraceTogether. The proportion of respondents believing that TraceTogether collects a lot of personal data increased concomitantly with the increase in adoption rates. Although the ease of using TraceTogether did not emerge as a factor in cohort 3, the impression on its ease of use improved across the cohorts (over time) as the adoption rate increased.

The responses on liberal individualism were mixed among respondents. Approximately 40% of the respondents agreed that they were only protecting others when they use TraceTogether. One-fifth of respondents took a neutral stance towards the notions of the common good, while two-fifths (40%) stood on the opposing ends of the spectrum on liberal individualism and altruism, respectively. Two-third of respondents across all cohorts agreed that they would use TraceTogether only if it benefited them, although this variable did not load well in the ‘ Liberal individualism’ factor in cohorts 2 and 3.

### Determinants of TraceTogether's acceptance

The final models for each of the three cohorts are shown in Table 2. Respondents who ‘perceived TraceTogether as useful and necessary’ had higher likelihood of accepting TraceTogether ([cohort] AOR (95% CI) [1] 2.77 (2.29–3.34); [2] 2.04 (1.61–2.58); [3] 2.30 (1.62–3.25)). Those with ‘Concerns about personal data collected by TraceTogether’ had lower likelihood of accepting TraceTogether ([cohort] AOR (95% CI) [1] 0.68 (0.60–0.78); [2] 0.62 (0.52–0.75); [3] 0.53 (0.40–0.70)). Only respondents in cohort 1 who ‘valued their personal and loved ones’ health during the COVID-19 pandemic’ had slightly higher likelihood of being willing to use TraceTogether. Individualistic notions do not have a significant impact on the acceptance of TraceTogether across all cohorts.

**Table 2.** Multivariable analysis of factors associated with the acceptance of TraceTogether (reference: unwilling/unsure about using TraceTogether)

	Cohort 1 (Jul–Oct 2020)		Cohort 2 (Nov–Dec 2020)		Cohort 3 (Jan–Feb 2021)	
Model variable	AOR (95% CI)	*P*-value	AOR (95% CI)	*P*-value	AOR (95% CI)	*P*-value
Factors from PCA						
Perceived TraceTogether as useful and necessary	2.77 (2.29–3.34)	**<0**.**001**	2.04 (1.61–2.58)	**<0**.**001**	2.30 (1.62–3.25)	**<0**.**001**
Values personal and loved ones' health	1.16 (1.02–1.32)	**0**.**026**	–		–	
Concerns about personal data collected by TraceTogether	0.68 (0.60–0.78)	**<0**.**001**	0.62 (0.52–0.75)	**<0**.**001**	0.53 (0.40–0.70)	**<0**.**001**
Liberal individualism	0.88 (0.77–1.01)	0.071	0.87 (0.72–1.05)	0.137	–	
Individual variables^a^						
I think people who are important to me (i.e. family members, spouse, friends) would recommend the use of the TraceTogether app/token [Q10].	1.62 (1.39–1.88)	**<0**.**001**	1.52 (1.24–1.87)	**<0**.**001**	1.68 (1.25–2.27)	**0**.**001**
I think the TraceTogether app/token is currently used by a lot of people [Q11].	1.35 (1.17–1.56)	**<0**.**001**	1.71 (1.40–2.08)	**<0**.**001**	1.49 (1.09–2.04)	**0**.**012**
I believe that my data collected by the TraceTogether app/token is secure [Q17].	1.26 (1.06–1.49)	**0**.**008**	1.44 (1.13–1.84)	**0**.**003**	1.67 (1.23–2.27)	**0**.**001**
I would use the TraceTogether app/token only if it benefits me [Q19].	–		0.80 (0.70–0.92)	**0**.**002**	–	
Demographics^b^						
>50 years of age	1.31 (1.03–1.68)	**0**.**031**	–		–	

Significance is *p* < 0.05 for bolded values.

a Refer to Supplementary Table S1 for the Likert scale questions.

b All demographics variables are binary.

The likelihood of accepting TraceTogether is higher among respondents who think that the ‘people important to them would recommend the use of TraceTogether’ ([cohort] AOR (95% CI) [1] 1.62 (1.39–1.88); [2] 1.52 (1.24–1.87); [3] 1.68 (1.25–2.27)) and if ‘TraceTogether is used by many people’ ([cohort] AOR (95% CI) [1] 1.35 (1.17–1.56); [2] 1.71 (1.40–2.08); [3] 1.49 (1.09–2.04)).

Respondents had increasing likelihood of accepting TraceTogether over time if they believe that the data collected by the TraceTogether are secure ([cohort] AOR (95% CI) [1] 1.26 (1.06–1.49); [2] 1.44 (1.13–1.84); [3] 1.67 (1.23–2.27)). Respondents in cohort 2 were less likely to accept TraceTogether if they felt that it only benefits themselves (AOR (95% CI) 0.80 (0.70–0.92)) and older adults (>50 years old) in cohort 1 are more likely to accept TraceTogether (AOR (95% CI) [cohort 1] 1.31 (1.03–1.68)).

### Determinants of TraceTogether's adoption

The final models for each of the three cohorts are shown in Table 3. Respondents who ‘perceived TraceTogether as useful and necessary’ had higher likelihood of adopting TraceTogether ([cohort] AOR (95% CI) [1] 1.31 (1.13–1.52); [2] 1.22 (1.03–1.45); [3] 1.38 (1.05–1.81)) while respondents with ‘Concerns about personal data collected by TraceTogether’ had lower likelihood of adopting TraceTogether ([cohort] AOR (95% CI) [1] 0.69 (0.61–0.77); [2] 0.73 (0.64–0.84); [3] 0.74 (0.59–0.93)). Respondents in all cohorts who found TraceTogether easy to use had higher likelihood of adopting TraceTogether ([cohort] AOR (95% CI) [1] 1.77 (1.57–2.01); [2] 2.51 (2.14–2.95); [3-Q9] 1.84 (1.15–2.94)) and respondents in cohort 1 who had individualistic notions had lower likelihood of adopting TraceTogether (AOR (95% CI) 0.87 (0.78–0.98)).

**Table 3.** Multivariable analysis of factors associated with the adoption of TraceTogether (ref: not using TraceTogether)

	Cohort 1 (Jul–Oct 2020)		Cohort 2 (Nov–Dec 2020)		Cohort 3 (Jan–Feb 2021)	
Model variable	AOR (95% CI)	*P*-value	AOR (95% CI)	*P*-value	AOR (95% CI)	*P*-value
Factors from PCA						
Perceived TraceTogether as useful and necessary	1.31 (1.13–1.52)	**<0**.**001**	1.22 (1.03–1.45)	**0**.**024**	1.38 (1.05–1.81)	**0**.**019**
Concerns about personal data collected by TraceTogether	0.69 (0.61–0.77)	**<0**.**001**	0.73 (0.64–0.84)	**<0**.**001**	0.74 (0.59–0.93)	**0**.**009**
Ease of use	1.77 (1.57–2.01)	**<0**.**001**	2.51 (2.14–2.95)	**<0**.**001**	–	
Liberal individualism	0.87 (0.78–0.98)	**0**.**019**	–		0.86 (0.69–1.08)	0.205
Individual variables^a^						
I think people who are important to me (i.e. family members, spouse, friends) would recommend the use of the TraceTogether app/token [Q10].	1.31 (1.13–1.51)	**<0**.**001**	1.41 (1.18–1.68)	**<0**.**001**	1.37 (1.03–1.81)	**0**.**030**
I think the TraceTogether app/token is currently used by a lot of people [Q11].	1.67 (1.48–1.90)	**<0**.**001**	1.55 (1.33–1.81)	**<0**.**001**	1.70 (1.29–2.25)	**<0**.**001**
It is not my concern if those I have been in contact with fall ill or die from COVID–19 [Q23].	0.87 (0.78–0.97)	**0**.**009**	–		–	
I would use the TraceTogether app/token only if it benefits me [Q19].	–		0.85 (0.76–0.94)	**0**.**002**	–	
I need someone (e.g. a family member) to teach me/show me how to use the TraceTogether app/token [Q8].	–		–		0.56 (0.44–0.69)	**<0**.**001**
I think TraceTogether app/token is/will be easy to use [Q9].	–		–		1.84 (1.15–2.94)	**0**.**011**
Demographics^b^						
>50 years of age	–		1.89 (1.42–2.52)	**<0**.**001**	2.39 (1.49–3.85)	**<0**.**001**
Employed	–				–	
Tertiary educated	1.47 (1.16–1.86)	**0**.**002**	–		–	

Significance is *p* < 0.05 for bolded values.

a Refer to Supplementary Table S1 for the Likert scale questions.

b All demographic variables are binary.

The likelihood of adopting TraceTogether is higher among respondents who think that the ‘people important to them would recommend the use of TraceTogether’ ([cohort] AOR (95% CI) [1] 1.31 (1.13–1.51); [2] 1.41 (1.18–1.68); [3] 1.37 (1.03–1.81)) and respondents who think that ‘TraceTogether is used by many people’ ([cohort] AOR (95% CI) [1] 1.67 (1.48–1.90); [2] 1.55 (1.33–1.81); [3] 1.70 (1.29–2.25)).

Respondents in cohort 1 who were ‘not concerned about people who may fall ill or die from COVID-19 due to them’ were less likely to adopt TraceTogether (AOR [1] (95% CI) 0.87 (0.78–0.97)). Respondents in cohort 2 were also less likely to adopt TraceTogether if they ‘think that it only benefits themselves’ (AOR [2] (95% CI) 0.85 (0.76–0.94)) and likewise for respondents in cohort 3 if they ‘needed someone to teach them how to use TraceTogether’ (AOR [3] (95% CI) 0.56 (0.44–0.69)).

Older adults (>50 years old) in cohorts 2 and 3 ([cohort] AOR (95% CI) [2] 1.98 (1.46–2.70); [3] 2.39 (1.49–3.85)), employed respondents in cohort 2 (AOR 1.37 (1.01–1.86)) and tertiary educated respondents in cohort 1 (AOR (95% CI) 1.41 (1.10–1.80)) were more likely to adopt TraceTogether.

## Discussion

We assessed the determinants of the acceptance and adoption of Singapore's national DCT tool – TraceTogether – across the different periods when new policy measures were implemented to increase its adoption, in response to the evolving COVID-19 pandemic. The attitudes and perceptions of health technology adoption among TraceTogether's users are complex under the circumstances of the COVID-19 pandemic and policies that necessitate its adoption. Hence, the stratified analysis enabled us to assess the attitudes and perceptions of TraceTogether concomitantly with the various policy implementations that precipitated the uptake of TraceTogether.

Respondents were more likely to accept and adopt TraceTogether if they perceived it as useful and necessary during the COVID-19 pandemic. Likewise, the perceived lack of benefit was found to be a barrier to DCT app uptake in a Swiss study [25]. The perceived benefit of the app is important in fostering voluntary acceptance of the tool [26]. However, voluntary adoption depends on other factors such as the severity of the pandemic, the users' adaptability to lifestyle changes and whether the perceived benefits of using the DCT tool outweigh its concerns [27].

Studies have cited concerns on the privacy and security of DCT tools as the main barriers to its adoption [16, 25]. Although respondents with concerns about their personal data collected by TraceTogether were less likely to accept and adopt TraceTogether, the likelihood of accepting TraceTogether due to belief in its data security increased over time. The stronger belief in TraceTogether's data security over time likely stemmed from increased user knowledge and adaptability to the tool [9]. Since DCT tools collect a large amount of data, trust in the government's ability to safeguard data is crucial in fostering the acceptance of such tools [28].

Another factor that could influence the acceptance and adoption of TraceTogether is the notion of the common good. As expected, respondents who valued liberal individualism were less likely to accept and adopt TraceTogether [23]. Other studies have also found that individuals with a higher level of altruism were more likely to comply with pandemic measures [29–31]. Altruistic behaviours are likely nuanced and may amplify as the pandemic worsens [32]. Hence, communicating the severity of the pandemic may encourage compliance with pandemic measures that can promote social good.

Apart from the policy measures that require the use of TraceTogether, influence from family members and widespread popularity may nudge hesitant individuals to adopt the new technology [20]. We found that the injunctive (what people approve) and descriptive (what people do) social norms were positive significant determinants of the acceptance and adoption of TraceTogether throughout the entire study period. References from relevant people create a system that can foster a sense of belonging in the adoption of a health behaviour or technology [33]. In technology adoption, additional barriers to its accessibility and usability should be addressed to increase the adoption rate.

One essential measure to increase the uptake of TraceTogether was to bridge the digital divide among people with and without access to or knowledge of use of smartphones and the Internet. The tertiary educated and employed were more likely to be early adopters of TraceTogether, while older adults had a higher likelihood of acceptance but low adoption rates at the earlier period of TraceTogether's implementation. Older adults were more likely to adopt TraceTogether only after Bluetooth tokens were distributed to the population, although the increase in adoption rates was likely a combination of other promotional and mandatory measures to accelerate the population uptake rates. Since the ease of use of TraceTogether was mainly associated with adoption, the increased likelihood of adoption over time due to this factor could have been a combination of the effect of adaptability to the tool under the conditions of mandatory adoption and its ease of use. Notably, the acceptance or adoption of TraceTogether was not different between gender.

Limitations of the study include our inability to generalise the study over the entire population. Although respondents were limited to the patients and visitors of two hospital outpatient clinics, the study team purposively recruited a diverse profile of respondents to minimise biases that would substantially skew the population profile. We also could not assess the changes in perceptions of the same respondent longitudinally over time, as the study was cross-sectional in nature. Further work could explore the notions of the common good in other populations on the acceptance of health technology in response to large-scale infectious diseases outbreaks. Future qualitative studies could also provide more in-depth understanding of the motivations that influence the acceptance and adoption of new technologies under the circumstances of a pandemic.

## Conclusion

In conclusion, the perceived necessity, injunctive social norms and descriptive social norms were major determinants of the acceptance and adoption of a national DCT tool during the course of the COVID-19 pandemic. On the other hand, concerns about personal data collected by TraceTogether and liberal individualism were barriers to its adoption. Policy measures to increase the uptake of the tool bridged the digital divide and accelerated its adoption. However, good public communications are crucial to address the barriers to acceptance to improve voluntary uptake and widespread adoption.

## Data Availability

The final dataset is partially available on request.

## Supplementary material

For supplementary material accompanying this paper visit https://doi.org/10.1017/S0950268822000401. click here to view supplementary material
