# Supplementary material for: Determinants of the acceptance and adoption of a digital contact tracing tool during the COVID-19 pandemic in Singapore
Source: Epidemiol Infect. 2022 Mar 2;150:e54. doi: 10.1017/S0950268822000401 (PMC8914141; doi:10.1017/S0950268822000401)
Supplement: Supplementary file 1 [file S0950268822000401sup001.docx]

**Supplementary files**

**Table S1: Likert-scale items to assess the determinants of acceptance and adoption of TraceTogether**

| **S/N** | **Likert scale statements** | **Constructs on health technology adoption** | **Survey options** |
| --- | --- | --- | --- |
| 1 | Using a contact tracing digital tool such as TraceTogether is a good idea. | Intention to use | Strongly Agree (5)  Agree (4)  Neither  Agree nor Disagree (3)  Disagree (2)  Strongly Disagree (1) |
| 2 | I don't see any problem with using a contact tracing digital tool such as TraceTogether. |  |  |
| 3 | I believe that using the TraceTogether app/token will protect me from COVID-19. | Perceived usefulness |  |
| 4 | I believe that TraceTogether will save me time if I am interviewed for contact tracing. |  |  |
| 5 | I think that TraceTogether will be useful for everyone, for contact tracing. |  |  |
| 6 | I think that TraceTogether will be useful for the young, the elderly, and those with dementia, for contact tracing. |  |  |
| 7 | I think that TraceTogether is useful in helping Singapore to control COVID-19. |  |  |
| 8 | I need someone (e.g., a family member) to teach me/show me how to use the TraceTogether app/token. | Ease of Use |  |
| 9 | I think TraceTogether app/token is/will be easy to use. |  |  |
| 10 | I think people who are important to me (i.e., family members, spouse, friends) would recommend the use of the TraceTogether app/token. | Injunctive social norm |  |
| 11 | I think the TraceTogether app/token is currently used by a lot of people. | Descriptive social norm |  |
| 12 | I believe that digital contact tracing technology (e.g., TraceTogether) collects lots of personal data about me. | Trust in Technology |  |
| 13 | I am concerned with my location being tracked by the TraceTogether app/token. |  |  |
| 14 | I am afraid of being infected with COVID-19. | Health valuation |  |
| 15 | It is important that I do not infect my loved ones with COVID-19. |  |  |
| 16 | If I have been a contact of someone confirmed with COVID-19, I would want to know this as soon as possible. |  |  |
| 17 | I believe that my data collected by the TraceTogether app/token is secure. | Perceived security of technology |  |
| 18 | I believe that I am protected by the law should there be a data breach by TraceTogether. |  |  |
| 19 | I would use the TraceTogether app/token only if it benefits me. | Notions of the common good |  |
| 20 | People (including my contacts) may still fall ill and die, even if they had used a digital contact tracing technology. |  |  |
| 21 | I value my individual freedom above the good of the community as a whole. |  |  |
| 22 | I am only protecting others when I use a digital contact tracing tool such as TraceTogether. |  |  |
| 23 | It is not my concern if those I have been in contact with fall ill or die from COVID-19. |  |  |

**Table S2a: Model selection for cohort 1 (Jul 2020 – Oct 2020)**

| **Model** | **Variables include in the model** | **Akaike Information Criteria** | |
| --- | --- | --- | --- |
|  |  | **Adoption** | **Acceptance** |
| M1 | All 5 factors from the PCA | 2024.29 | 1666.59 |
| M2 | M1-Factor 2 | 2022.66 | - |
| M3 | M2+Q10+Q11+Q14+Q17+Q18+Q23* | 1980.38 | - |
| M4 | M2+Q10+Q11+Q23 | 1975.87 | - |
| M5 | M4+Employment status | 1980.19 | - |
| **M6** | **M4+Education level** | **1975.93** | - |
| M7 | M1-Factor 4 | - | 1665.29 |
| M8 | M8+Q10+Q11+Q17* | - | 1621.18 |
| **M9** | **M9+Age** | - | **1621.28** |

*The individual question in the models corresponds to the questions in Table S1.

M6 and M9 were chosen as the final models to assess the determinants for the adoption and acceptance of a DCT tool during the COVID-19 pandemic, respectively. Models with the variables of interest, with the lowest Akaike Information Criteria preferred, were chosen as final. Age and educational level were all coded into binary variables.

**Table S2b: Model selection for cohort 2 (Nov 2020 – Dec 2020)**

| **Model** | **Variables include in the model** | **Akaike Information Criteria** | |
| --- | --- | --- | --- |
|  |  | **Adoption** | **Acceptance** |
| M1 | All 5 factors from the PCA | 1316.09 | 799.77 |
| M2 | M1-Factor 4 | 1314.46 | - |
| M3 | M2+Q10+Q11* | 1305.79 | - |
| M4 | M3+Q19* | 1321.63 | - |
| M5 | M4-Factor 2 | 1320.46 | - |
| **M6** | **M5+Age** | **1330.52** | - |
| M7 | M1 -Factor 2 -Factor 5 | - | 859.12 |
| **M8** | **M7+ Q10+Q11+Q17+Q19*** | - | **823.66** |

*The individual question in the models corresponds to the questions in Table S1.

M6 and M8 were chosen as the final models to assess the determinants for the adoption and acceptance of a DCT tool during the COVID-19 pandemic, respectively. Models with the variables of interest (and statistically significant) were chosen over the Akaike Information Criteria. Age was coded as a binary variable.

**Table S2c: Model selection for cohort 3 (Jan 2021 – Feb 2021)**

| **Model** | **Variables include in the model** | **Akaike Information Criteria** | |
| --- | --- | --- | --- |
|  |  | **Adoption** | **Acceptance** |
| M1 | All 5 factors from the PCA | 575.35 | 452.580 |
| M2 | M1 -Factor 2 | 574.88 | 451.050 |
| M3 | M2+Q8+Q9+Q10+Q11* | 570.98 |  |
| **M4** | **M3+Age** | **570.58** |  |
| M5 | M2+Q10+Q11* | - | 448.13 |
| M6 | M5 -Factor 3 | - | 446.88 |
| **M7** | **M6+Q16+Q17*** | - | **449.44** |

*The individual question in the models corresponds to the questions in Table S1.

M4 and M7 were chosen as the final models to assess the determinants for the adoption and acceptance of a DCT tool during the COVID-19 pandemic, respectively. Models with the variables of interest (and statistically significant) were chosen over the Akaike Information Criteria. Age was coded into a binary variable.
